# Holistic assessment of the microbiome dynamics in the substrates used for commercial champignon (*Agaricus bisporus*) cultivation

**DOI:** 10.1111/1751-7915.13639

**Published:** 2020-07-27

**Authors:** Jaime Carrasco, Carlos García‐Delgado, Rebeca Lavega, María L. Tello, María De Toro, Víctor Barba‐Vicente, María S. Rodríguez‐Cruz, María J. Sánchez‐Martín, Margarita Pérez, Gail M. Preston

**Affiliations:** ^1^ Department of Plant Sciences University of Oxford S Parks Rd Oxford OX1 3RB UK; ^2^ Centro Tecnológico de Investigación del Champiñón de La Rioja (CTICH) Autol Spain; ^3^ Departamento de Geología y Geoquímica Universidad Autónoma de Madrid Madrid Spain; ^4^ Institute of Natural Resources and Agrobiology of Salamanca (IRNASA‐CSIC) Salamanca Spain; ^5^ Plataforma de Genómica y Bioinformática Centro de Investigación Biomédica de La Rioja (CIBIR) Logroño Spain

## Abstract

Microorganisms strongly influence and are required to generate the selective substrate that provides nutrients and support for fungal growth, and ultimately to induce mushroom fructification under controlled environmental conditions. In this work, the fungal and bacterial microbiota living in the different substrates employed in a commercial crop (compost phase I, II and III, flush 1 and 2, and casing material on day 1, 6 and 8 after compost casing and during flush 1 and 2) have been characterized along the different stages of cultivation by metataxonomic analysis (16S rRNA and ITS2), analysis of phospholipid fatty acid content (PLFAs) and RT‐qPCR. Additionally, laccase activity and the content of lignin and complex carbohydrates in compost and casing have been quantified. The bacterial diversity in compost and casing increased throughout the crop cycle boosted by the connection of both substrates. As reflected by the PLFAs, the total living bacterial biomass appears to be negatively correlated with the mycelium of the crop. *Agaricus bisporus* was the dominant fungal species in colonized substrates, displacing the pre‐eminent Ascomycota, accompanied by a sustained increase in laccase activity, which is considered to be a major product of protein synthesis during the mycelial growth of champignon. From phase II onwards, the metabolic machinery of the fungal crop degrades lignin and carbohydrates in compost, while these components are hardly degraded in casing, which reflects the minor role of the casing for nourishing the crop. The techniques employed in this study provide a holistic and detailed characterization of the changing microbial composition in commercial champignon substrates. The knowledge generated will contribute to improve compost formulations (selection of base materials) and accelerate compost production, for instance, through biotechnological interventions in the form of tailored biostimulants and to design environmentally sustainable bio‐based casing materials.

## Graphical abstract

The techniques employed in this study provide a holistic and detailed characterization of the changing microbial composition in commercial champignon substrates. The knowledge generated will contribute to improve formulations through biotechnological interventions.

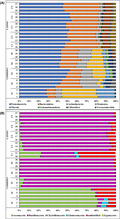

## Introduction

Mushroom production is a well‐known example of the circular economy that employs by‐products from agricultural and farming activities such as wheat straw or chicken manure to generate nutritious and protein‐rich food (Roncero‐Ramos and Delgado‐Andrade, 2017; Carrasco *et al*., 2018a). Therefore, cultivated mushrooms can provide an important contribution to the rising demand for sustainable protein from eco‐friendly sources with lower environmental impact than animal products (Poore and Nemecek, 2018; Springmann *et al*., 2018).

According to Royse *et al*. (2017), the champignon or button mushroom (*Agaricus bisporus*) is among the most cultivated mushroom species and Europe has a key role in the sector, accounting for over 30% of total market share with an annual production of 1.37 billion kg (Royse *et al*., 2017). The commercial cultivation of champignon is a highly controlled agricultural activity, done indoors, in the dark and during the entire year, providing stable production without relying on favourable outdoors climatic conditions or seasonality. However, as heterotrophic organisms, cultivated fungi require nutritional input from lignocellulosic substrates that are generated through a number of composting steps (Carrasco *et al*., 2018b). Additionally, substrates that have been inoculated with the mycelium of the crop require casing material, commonly peat‐based, with optimal physicochemical and microbiological characteristics to induce mushroom fructification (Pardo‐Giménez *et al*., 2017; Carrasco *et al*., 2019).

Metataxonomic analysis conducted through NGS does not rely on enrichment or isolation, and so it is possible to work with crude environmental samples, offering a new spectrum of possibilities to characterize the microbe‐rich environmental niche in which *A. bisporus* grows and develops. Recent publications have reported the role of bacterial strains during the aerobic solid fermentation process required to produce the selective substrate employed in champignon cultivation (Kertesz and Thai, 2018; Vieira and Pecchia, 2018), the structure and dynamics of the bacterial and fungal community associated with the mycelium of the crop during cultivation (Kertesz and Thai, 2018; McGee *et al*., 2017a, 2017b), and the bacterial and fungal profiling and dynamic succession of the microbiota that cohabits with the crop in the casing material (Carrasco *et al*., 2019; Yang *et al*., 2019). In order to deepen our knowledge of the interactions of champignon and the surrounding microbiota, the objective of this study was to evaluate the composition and progression of the fungal and bacterial microbiome living in different commercial substrates employed in commercial champignon cultivation.

Longitudinal analysis of commercial compost (phase I, phase II and phase III, flush 1 and flush 2) and casing materials (at day 1 after compost casing, day 6, day 8, flush 1 and flush 2) has been conducted qualitatively through metataxonomic analysis (NGS; 16S rRNA and ITS2 metagenomics), and quantitatively through analysis of phospholipid fatty acid content (PLFAs) and RT‐qPCR. Specific PLFAs have been used as biomarkers to quantify bacterial and fungal microbiota (Vos *et al*., 2017a).

*Agaricus bisporus* secretes a range of extracellular enzymes to degrade the lignin (aromatic complex) and complex carbohydrates, cellulose and hemicellulose (xylan) present in crop substrates, including cellulases, laccase, peroxidases and xylanases (hemicelluloses) (Jurak *et al*., 2014, 2015; Kabel *et al*., 2017). Among the extracellular enzymes, laccase is considered to be a major product of protein synthesis during the mycelial growth of champignon (Kabel *et al*., 2017) and therefore is a suitable marker for the vegetative growth of the fungus (Vos *et al*., 2017a). In order to investigate the potential correlations between microbiome composition and the metabolic activity of the crop, the metabolic behaviour of *A. bisporus* was studied through quantitative analyses of both laccase activity and the relative content of lignin and carbohydrates in compost and casing.

## Results

### Metataxonomic analysis

**Fig. 1:**
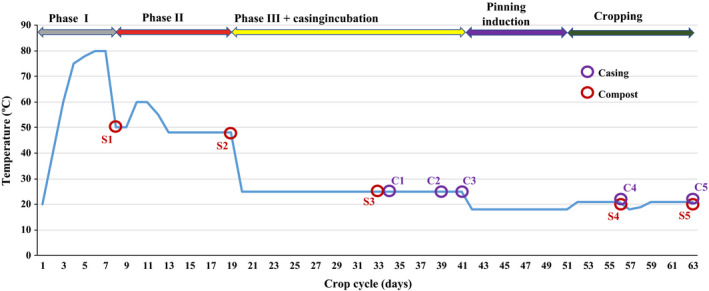
Samples taken along the process of composting and mushroom cultivation from a commercial compost yard and commercial crop located in La Rioja, Spain: compost (S1: phase I, S2: phase II and S3: phase III, and S4: flush 1 and S5: flush 2) and casing materials (C1: day 1, C2: day 6, C3: day 8, C4: flush 1 and C5: flush 2).

The commercial cultivation of mushroom requires the generation of a selective substrate through a three‐step composting process, including mycelium inoculation to create the phase III compost (Vieira and Pecchia, 2018). However, a compost fully colonized by the *A. bisporus* mycelium barely produces sporophores and it must be topped with a casing material to induce fructification and obtain commercially viable production (Carrasco *et al*., 2018b). Samples were taken in triplicate at five different points along the crop cycle, from compost (S1–S5) and casing (C1–C5) from a commercial growing unit (Fig. 1).

**Fig. 2:**
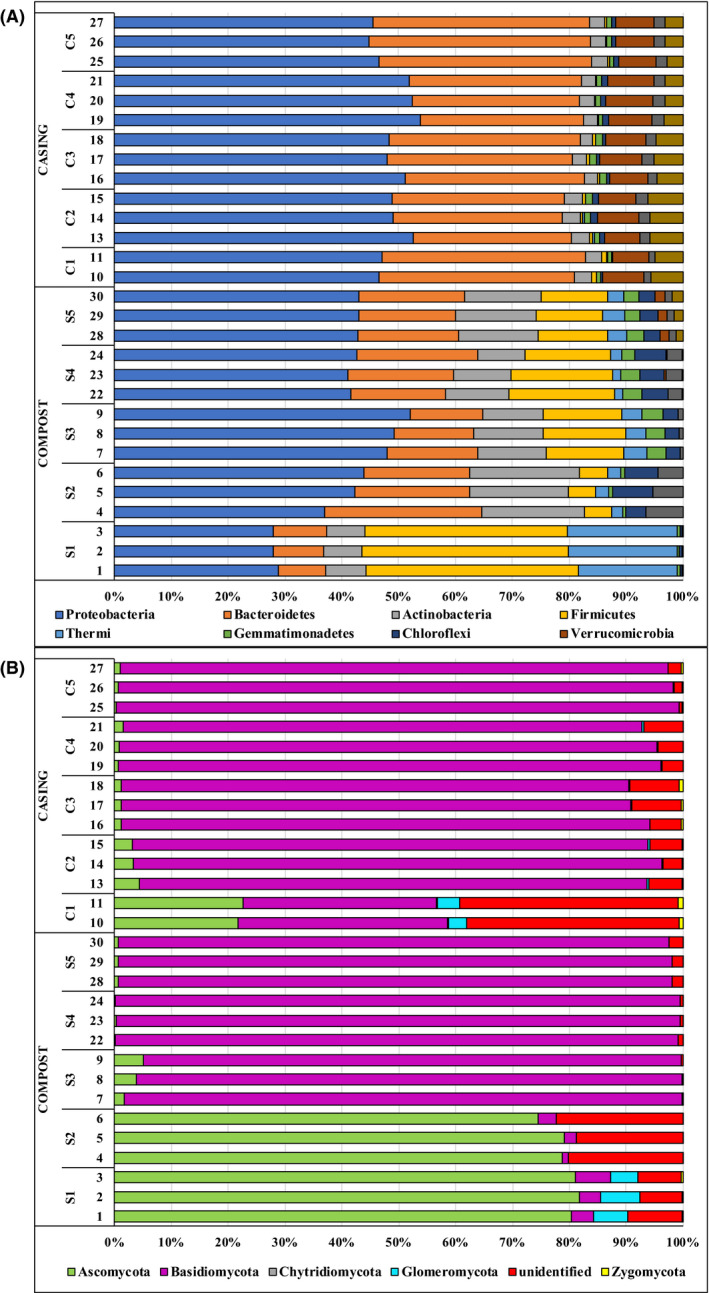
Relative abundance of OTUs at the level of phylum showing the composition and dynamics of the microbiome along the crop cycle. A. 16S rRNA (10 most abundant phyla; >87% OTUs per sample). B. ITS (100 % OTUs per sample). Taxa have been merged based on the sum of their counts across samples and groups. Features with fewer than 10 counts and non‐assigned or unidentified OTUs have been removed to help the visualization of major trends. Compost (S1: phase I, S2: phase II and S3: phase III, and S4: flush 1 and S5: flush 2) and casing materials (C1: day 1, C2: day 6, C3: day 8, C4: flush 1 and C5: flush 2).

To investigate the dynamic changes in microbiome composition that occur during mushroom cultivation, we performed a metagenomic analysis of bacteria and fungi present in both the compost and casing along a commercial cropping cycle, as summarized in Figure 2. The composition of the bacterial and fungal microbiome in compost/nutritive substrate (S1: phase I, S2: phase II and S3: phase III, and S4: flush 1 and S5: flush 2) and casing materials (C1: day 1, C2: day 6, C3: day 8, C4: flush 1 and C5: flush 2) was determined by the characterization of libraries based on the amplification of V3–V4 16S rRNA (bacteria) and ITS2 (fungi). After filtering and quality control, 1871042 reads, with average count per sample equal to 64518 reads, remained for 29 V3–V4 16S rRNA amplified region from 5 compost sample groups (S1–S5) and 5 casing sample groups (C1–C5) (sample 12, C1, 16S rRNA was excluded due to the reduced quality of reads achieved for this replicate), and 4 453 497 reads, with average count per sample equal to 148 450 reads, remained for the 30 ITS2 amplified region from 5 compost sample groups (S1–S5) and 5 casing sample groups (C1–C5) (Table S1). Sixty‐eight genera and 24 phyla of bacteria [1111 OTUs (Operational Taxonomic Units)] and 18 genera and 5 phyla of fungi (194 OTUs identified) were assigned across all samples. More than 99% of OTUs detected were assigned at the level of genus for the fungal microbiome. However, between 51% and 78% of detected OTUs could not be assigned to the level of bacterial genus (Data S1 – Abundance OTUs.xlsx). The replicates for each group of samples, both for bacteria and for fungi, show only minor differences, which supports the reproducibility of the methodology applied (Fig. 2).

With respect to the taxonomic relative abundance, phase I was characterized by the predominance of bacteria belonging to the phyla *Firmicutes*, *Proteobacteria* and *Thermi* and fungi from the *Ascomycot*a phylum, with a comparatively consistent fungal community structure in phase II (Fig. 2A and B). However, the bacterial community in phase II was found to show a drastic decrease in *Firmicutes* and *Thermi*, which were replaced by *Proteobacteria*, *Bacterioidetes* and *Actinobacteria* as dominant phyla. The bacteria belonging to the thermophilic genus *Thermus* were dominant in phase I, and the actinobacteria genera, *Actinomadura,* became the most abundant in phase II (Data S1 – Abundance OTUs.xlsx).

Colonization of compost by the mycelium of *A. bisporus* (phase III) resulted in an increase in *Firmicutes* and *Gemmatimonadetes* (*Chelatococcus*, *Themofibia* and *Thermus* were the most abundant genera) (Fig. 2A), while the fungal community unsurprisingly showed a dramatic change with the predominance of *Basidiomycota* (*Agaricus*), which replaced the pre‐eminent *Ascomycota* community. *Schyzophillum* spp. was the most abundant fungal species during the composting stages (S1, S2), but its relative abundance dropped gradually in compost from phase III, when *A. bisporus* became dominant, to the end of the crop (Fig. 2B). Along the process of cultivation, the relative abundance of *Pseudomonas* increased to become the second most abundant in the first flush (S4) and the most abundant by the end of the cycle (S5).

The *Proteobacteria* genus *Sphingobium* and the *Verrucomicrobia* genus *Prosthecobacter* were dominant in non‐colonized casing; however, the presence of *A. bisporus*, from C2–C5, established a stable bacterial microbiome in which the *Bacterioidetes* genus of *Flavobacterium* and the *Proteobacteria* genus of *Devosia* were the two most abundant. It is also important to note the increase in the relative abundance of the genus *Pseudomonas* observed in the casing along the process of cultivation, from residual in C1 to the fourth most abundant in C4. This same trend was observed for the predatory bacterial genus *Bdellovibrio*, the relative abundance of which even surpassed the relative abundance of *Pseudomonas* by the end of the trial (C5) (Data S1 – Abundance OTUs.xlsx). The fungal microbiome present in the casing was greatly influenced by the colonization of casing by *A. bisporus,* which replaced *Ascomycota*, *Glomeromycota* and *Zygomycota* as the predominant *Basidiomycota*, with no notable changes during the later stages of cultivation (Fig. 2B).

It is worth noting that an OTU corresponding to *Lecanicillium* sp. KYK00240 (GenBank Ac. n. AB378524), which shows 100% identity with the genome of *Lecanicillium fungicola* 150‐1 (GenBank Ac. n. FWCC01000001), causal agent of dry bubble disease (Banks *et al*., 2019), was identified in samples of phase II (S2), non‐colonized casing (C1), casing in second flush (C4) and casing in third flush (C5). Although this result must be interpreted with caution and needs to be confirmed by detection of parasite‐specific sequences, this suggests that hygiene measurements should be reinforced at commercial sites during compost and casing preparation to avoid early contamination of substrates and to prevent heavy outbreaks (Carrasco *et al*., 2019).

**Fig. 3:**
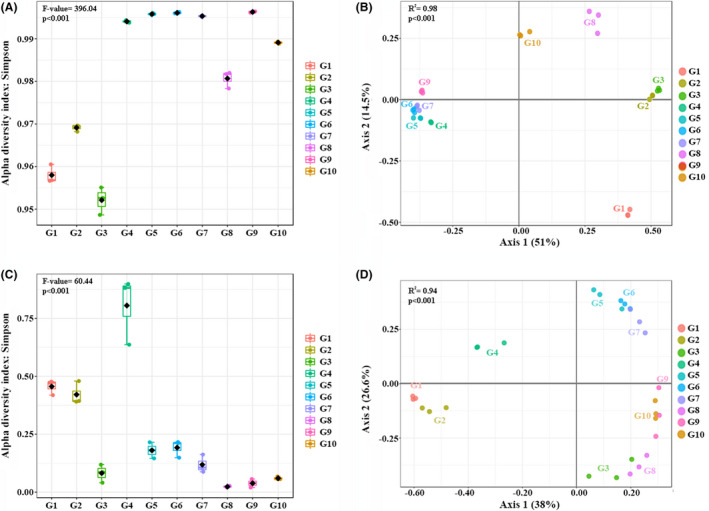
Alpha‐ and beta‐diversity analyses to compare the richness and abundance of OTUs among samples and groups. Alpha‐diversity analysis was performed for bacteria (A) and fungi (C) using evenness metrics (Simpson measure). Beta‐diversity analysis for bacteria (B) and fungi (D) was analysed using principal coordinates analysis (PCoA) with 2‐dimensional scaling. Compost (S1: phase I, S2: phase II and S3: phase III, and S4: flush 1 and S5: flush 2) and casing material (C1: day 1, C2: day 6, C3: day 8, C4: flush 1 and C5: flush 2).

Figure 3 shows the differences noted between samples for the bacterial community (Fig. 3A and B) and fungal community (Fig. 3C and D), as reflected by alpha‐ and beta‐diversity plots. Non‐colonized compost samples (S1 and S2) showed values of α‐diversity significantly lower for bacteria and fungi than non‐colonized casing (C1). The colonization of the casing by the mycelium of *A. bisporus* promoted a significant increase in the bacterial α‐diversity (α‐diversity refers to the species diversity and abundance in each group of samples; Carrasco *et al*., 2019) and a significant decrease in fungal α‐diversity (comparing C1 (non‐colonized casing material) with the rest of the casing samples).

Conversely, the α‐diversity in the compost colonized by *A. bisporus* (S3: phase III) was significantly lower than in phases I or II, with a noteworthy increase in bacterial α‐diversity after casing application (S4 and S5), which suggests that bacterial translation from casing to compost may occur (Fig. 3A and C). According to the PCoA (Fig. 3B and D), there was no correlation in β‐diversity (β‐diversity calculates the degree of similarity between habitats/group of samples; Carrasco *et al*., 2019) between the bacterial community in phase I (S1) and the community present in compost samples during production (S4 and S5), nor with the casing material (C1–C5 clustered together). The casing material showed significant differences in bacterial β‐diversity from phase II (S2) and phase III (S3) compost, while phase I compost differed from the compost samples collected during production (S4 and S5). With respect to fungal β‐diversity, the colonization of the substrates by the mycelium of the crop results in a clear differentiation of samples when comparing compost (S1–S2 vs S3–S4–S5) or casing (C1–C4 vs C5), with no correlation between the original fungal community of the compost (S1, S2) and the casing (C1).

The structure of the core bacterial microbiome was influenced by interactions between the two substrates, compost and casing, employed in mushroom cultivation as reflected (Figs S1–S3). Since S1, S2 and S3 corresponded to compost samples without contact with casing and C1 is casing raw material with no influence of compost, both materials interacted from C2 onwards. The influence of interaction was starkly demonstrated when observing firstly the compost samples covered with casing, in which the compost and casing samples shared a high proportion of OTUs during the first, S4, and second flush, S5 (Figs S1a and S2a), suggesting motility of casing inhabitants to the compost; and subsequently when regarding the OTUs shared by the colonized casing (C2), casing (C3), casing (C4) and casing F2 (C5) (Figs S1 and S2c), which implies motility of the bacterial community from the compost to the casing.

### PLFA analysis

The high structural diversity of PLFAs and their high biological specificity allows them to be used to quantitatively classify the microbiota of the sample into microbial groups (Vos *et al*., 2017a). PLFA analysis was therefore used to quantify the relative abundance of Gram‐ bacteria, Gram + bacteria, *Actinobacteria* and fungi along the cropping cycle.

**Fig. 4:**
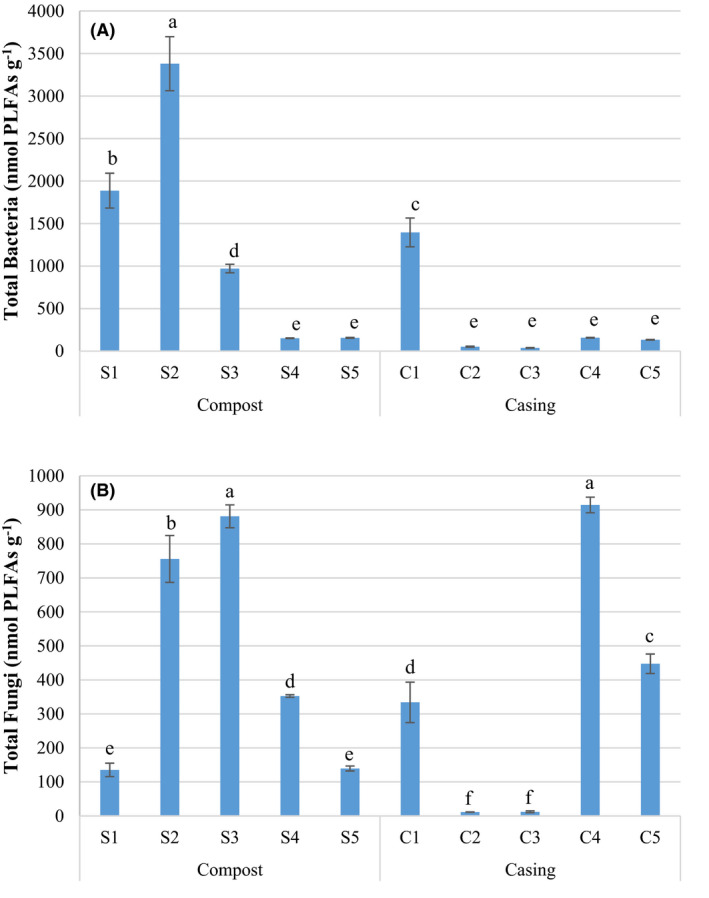
Biomass expressed in ng PLFAs g^−1^: (A) Total bacterial biomass (Gram‐, Gram + and *Actinobacteria*); (B) saprophytic fungal biomass. Bars indicate standard deviation (*n* = 4). Different letters indicate significant differences between samples (*P* < 0.05). Compost (S1: phase I, S2: phase II and S3: phase III, and S4: flush 1 and S5: flush 2) and casing materials (C1: day 1, C2: day 6, C3: day 8, C4: flush 1 and C5: flush 2). Per cent variability explained by each principal component is shown in parentheses after each axis legend.

The largest amount of bacterial biomass was found in the composting phases (S1 and ‐S2) and in casing not colonized by *A. bisporus* (C1) (Fig. 4a). Throughout the composting period, total bacterial biomass increased from phase I to phase II, and decreased in phase III (colonized by *Agaricus*). With respect to the casing (C1–C5), the total bacterial biomass dropped drastically during colonization by the *A. bisporus* mycelium (C2, C3) and increased again during the first flush (C4), remaining at similar levels in the second flush (C5). Throughout the production stage, the total bacterial biomass decreased in the compost (S4 and S5), and a similar trend was also observed in the casing material (C4 and C5) (Fig. 4a).

The most abundant bacterial categories in both substrate and casing samples were Gram + bacteria followed by Gram‐ bacteria and finally *Actinobacteria* (Fig. S4). Notably, different behaviours were observed for Gram‐ bacteria, Gram + bacteria and *Actinobacteria* during production (S4–S5 and C4–C5). In the casing (C4 and C5), the biomass of Gram‐ bacteria was higher than in the substrates (S4 and S5), while for Gram + bacteria and *Actinobacteria,* the opposite was observed.

Among the compost samples, there was a sharp increase in the total fungal biomass from phase I to phase II, while in the casing (C2, C3), there was a very sharp decline in the total fungal biomass after colonization by *A. bisporus*. However, a high fungal biomass was recovered from the casing during the first flush (C4), which decreased in the second flush (C5). With respect to phase III (S3), the compost undergoes a continuous decline in fungal biomass throughout the production cycle (S4 and S5), which was also observable in the casing (Fig. 4B).

**Fig. 5:**
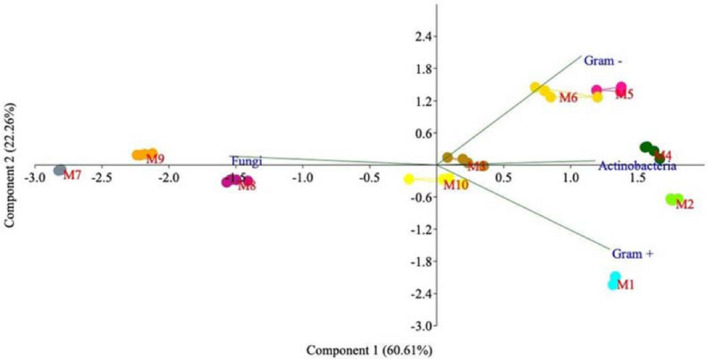
Principal components analysis (PCA) loading scores for Gram− and Gram+ bacteria, Actinobacteria and fungi. Compost (S1: phase I, S2: phase II and S3: phase III, and S4: flush 1 and S5: flush 2) and casing materials (C1: day 1, C2: day 6, C3: day 8, C4: flush 1 and C5: flush 2). Per cent variability explained by each principal component is shown in parentheses after each axis legend.

The proximity of the analytical replicates of each group of samples following PCA denotes the robustness of the method (Fig. 5).

As reflected in the PCA shown in Figure 5, *Actinobacteria* were positively related to component 1 (C2), while fungi were negatively related to C2, and their weight in component 2 (C1) was very low. This observation could be interpreted as indicating that when the proportion of fungi increased, that of Actinobacteria decreased. During cropping (S4, C4, C5), the microbial structure was clearly displaced towards a high proportion of fungi in both compost and casing, while in the samples obtained at the beginning of the crop, the fungal load was low and bacteria predominate.

During production, a decrease in fungal richness was observed both for casing (C4 and C5) and for the substrate (S4 and S5).

### RT‐qPCR and laccase activity

Real‐time PCR analysis was used to quantify bacterial and fungal biomass along the process of champignon cultivation. Additionally, quantification of laccase activity was used as a proxy for fungal biomass in both the compost and the casing (Table 1) (Vos *et al*., 2017a).

**Table 1:** Quantitative analysis of bacterial and fungal biomass using qPCR and laccase activity.

Sample		qPCR		Laccase activity
		Bacteria 16s rRNA (ng uL^‐1^)	Fungi 5.8Ss rRNA (ng uL^‐1^)	U gr^‐1^
Compost^a^	S1	52.2 ± 15.1ab^b^	2.6 ± 0.5e	14.5 ± 2.0e
	S2	45.7 ± 14.0bc	4.3 ± 0.3e	12.5 ± 3.3e
	S3	47.3 ± 7.1abc	39.4 ± 8.1c	186.9 ± 14.2b
	S4	61.0 ± 6.0a	67.4 ± 2.1a	269.5 ± 13.5a
	S5	38.5 ± 12.3bc	60.6 ± 1.1ab	59.9 ± 5.2c
Casing	C1	38.3 ± 8.5bc	3.0 ± 0.2e	7.4 ± 2.0e
	C2	43.3 ± 0.4bc	5.1 ± 0.2e	7.9 ± 2.9e
	C3	45.8 ± 0.6bc	33.4 ± 2.1c	6.5 ± 2.7e
	C4	36.6 ± 11.1c	15.3 ± 5.6d	29.4 ± 3.6d
	C5	50.6 ± 8.1abc	60.2 ± 6.7b	104.2 ± 2.3c

^a^ Compost (S1: phase I, S2: phase II and S3: phase III, and S4: flush 1 and S5: flush 2) and casing materials (C1: day 1, C2: day 6, C3: day 8, C4: flush 1 and C5: flush 2).

^b^ Values followed by equal letters in the columns (comparing each trial) are not significantly different according to post hoc Tukey's test (*P* < 0.05) and Kruskal–Wallis test and the Mann–Whitney (Wilcoxon) W test to compare medians at the 95% level, for non‐normal distributions.

The bacterial rRNA region quantified through RT‐qPCR showed relatively little variation throughout the cropping period. Conversely, the fungal DNA quantified by 5.8S rRNA amplification increased along the process of cultivation with significant differences observable between colonized and non‐colonized substrates. Compost and casing showed the highest values of fungal DNA by the end of the crop cycle in S4, S5 and C5 respectively (Table 1). These gradually growing values are attributable to the mycelium of the crop. This result was consistent with the laccase activity measured in both compost and casing. Laccase activity showed a sustained increase along the crop cycle with a notable decrease at the end of the crop cycle (S5), probably associated with the depletion of nutrients in spent mushroom compost or the degeneration of *A. bisporus* mycelium.

### Quantification of lignin and complex carbohydrates

During mushroom production lignocellulose is degraded through the action of a wide range of secreted enzymes, including cellulases, hemicellulases (xylanases), laccase and peroxidases (Kabel *et al*., 2017; Vos *et al*., 2017b). Here, we report the changes observed along the process of cultivation in the composition of the compost and casing plant cell wall structures, cellulose, hemicellulose and lignin.

**Fig. 6:**
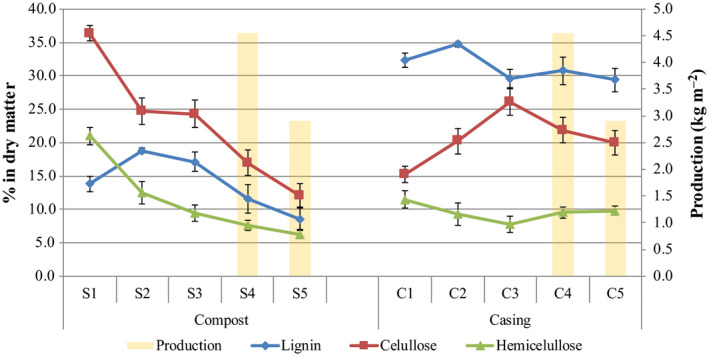
Relative dry matter content of lignin and carbohydrates, cellulose and hemicellulose, in the commercial substrates employed in champignon cultivation along the crop cycle. Error bars indicate standard deviation (*n* = 5). Stacked bars indicate mushroom production in first flush (S4 and C4) and second flush (S5 and C5) (compost, S1: phase I, S2: phase II and S3: phase III, and S4: flush 1 and S5: flush 2; casing, C1: day 1, C2: day 6, C3: day 8, C4: flush 1 and C5: flush 2).

The peat‐based casing used, which is composed of soil and sedimentary organic matter from degraded plant material and soil microorganisms (Younes and Grasset, 2018), was composed mainly of lignin (32.3%), the major component detected, and carbohydrates, cellulose (15.3%) and hemicellulose (11.5%) (Fig. 6). The relative dry matter composition of these main components remained fairly steady during the crop cycle, which suggests that the casing only plays a minor role in nourishing the crop.

However, the relative content of lignin, cellulose and hemicellulose in the commercial compost substrate varied during composting stages and during the cultivation stages. From S1 (phase I) to S2 (phase II), there appeared to be a significant depletion of cellulose (from relative content of 36.4% in S1 to 24.8% in S2) and hemicellulose (21.0% in S1 to 12.5% in S2), while the relative lignin content increased (13.8% in S1 to 18.8% in S2), indicating that lignin is not degraded during composting, which is consistent with previously reported data (Jurak *et al*., 2015). The colonization of the compost by the mycelium of the crop was reflected in a decrease in lignin and carbohydrates by the end of phase III (S3) and during cropping stages (S4 and S5). By the end of the crop cycle (S5), the lignin (8.6%), hemicellulose (6.3%) and cellulose (12.0%) in the compost were metabolized by almost 50% compared to phase II (18.8% of lignin, 12.5% of hemicellulose and 24.7% of cellulose respectively).

## Discussion

Mushrooms are a nutritious asset to include in our diets, which can be cultivated throughout the year without dependence on climate and promoting the circular economy (Grimm and Wösten, 2018). The microbiome understood as the microbial load cohabiting with the crop in the substrates employed in mushroom cultivation strongly influences the performance of the crop, for instance while driving the biological cues that trigger fructification (Carrasco and Preston, 2020). Here, we have examined the changes in microbiome composition that occur during mushroom cultivation, through qualitative and quantitative techniques, and characterized the substrate degradation and the enzymatic activity of *A. bisporus* by monitoring laccase as a marker alongside changes in substrate composition.

Throughout the composting process, a succession of bacterial and fungal communities transforms raw materials into a mature substrate for mushroom cultivation (Vieira and Pecchia, 2018; Cao *et al*., 2019). The depth of metagenome analysis achieved for bacteria in our analyses was lower than for fungi, in part due to the complexity of domain Bacteria compared with Eukarya and difficulties in culturing single organisms to generate genomic data (Hug *et al*., 2016). In our samples, phase I and phase II were dominated by microorganisms that are known to show an active cellulolytic profile. The ascomycete genera *Chaetomium* and *Thermomyces* are predominant in phase I compost, while *Scytalidium* sp. is the dominant species in phase I and II. *Thermomyces* is a genus of thermophilic fungus that produces cellulolytic, amylolytic, xylanolytic, proteolytic and lipolytic enzymes (Janda, 2005). One per cent of the reads in phase I compost matched the non‐cellulolytic fungus *Thermomyces lanuginosus*, a thermophilic fungus that produces extracellular hemicellulases involved in hemicellulose hydrolysis (Singh *et al*., 2003). The sequenced reads likely matching *Chaetomium* sp. AF‐18 (NCBI Ac. No JN168655) are consistent with the cellulolytic profile of the thermophilic soil fungus *Chaetomium thermophilum*, which has been shown to produce thermostable cellulase (Li *et al*., 2018). The most abundant OTUs in our compost samples corresponded to *Scytalidium* sp. (NCBI Ac. No HQ661373), in phase I (62.5%) and phase II (74.6%) but dropped from 2.8% in phase III to a residual presence during cultivation. It is worth noting that *Thermomyces* sp. ECS‐711 (Díaz‐Martínez *et al*., 2019) and *Scytalidium thermophilum* 15.8 (CBS671.88), *Chaetomium thermophilum* 209.3 and T49.6.5*, Chaetomium* sp.M4.7 (Straatsma *et al*., 1994), isolated mostly from mushroom compost, have been described as growth‐promoting thermophilic microorganisms to improve the mycelial growth of champignon (Díaz‐Martínez *et al*., 2019).

*Firmicutes, Proteobacteria* and *Thermi* were the most abundant bacterial phyla in phase I, with a prevalence of genera such as *Thermus, Shewanella, Arthrobacter, Providencia* or *Bacillus*. These microorganisms have been shown to contribute to the release of ammonia that softens the raw materials (Zhang *et al*., 2014; Vieira and Pecchia, 2018; Carrasco and Preston, 2020). By the end of phase II, bacterial living biomass significantly increased, especially Gram‐negative and *Actinobacteria*, with *Actinomadura, Chelatococcus* or *Steroidobacter* as dominant genera, and, as previously reported, a remarkable increase in the thermophilic genera *Pseudoxanthomonas* was noted (Vajna *et al*., 2012; Kertesz *et al*., 2016). One of the main roles assigned to the dominant microbiota in phase II is the removal of free ammonia that can adversely influence mushroom growth and favour the development of competitors/ parasites such as *Trichoderma* spp. (Carrasco and Preston, 2020).

With respect to the structural fibre content in commercial compost, our findings agree with those reported by Jurak *et al*. (2015), with significant degradation of carbohydrates observed during phase II, while lignin remains unaltered, and the subsequent action of the *A. bisporus* mycelium to degrade lignin, hemicellulose and cellulose during phase III and along the crop cycle. Mushrooms, as heterotrophic organisms, produce ligninolytic enzymes (such as laccase or Mn‐peroxidase) and hemicellulose‐ and cellulose‐degrading enzymes (such as xylanase or cellulase) that degrade compost structure (Sánchez, 2009; Kabel *et al*., 2017; Vos *et al*., 2017b).

In coincidence with the results previously noted by McGee *et al*. (2017b), *A. bisporus* became dominant in the colonized phase III compost and during cropping, replacing the pre‐eminent *Ascomycota* (Fig. 2B). As reflected by the fungal PLFAs and laccase activity, there was a marked decrease in the active fungal biomass from the first flush to the second by more than 50%, which is consistent with results reported by McGee *et al*. (2017b) when screening cDNA extracts from colonized compost.

Interestingly, the total bacterial biomass appears to be conditioned by the mycelium of the crop. The quantification of genomic DNA in environmental samples through RT‐qPCR does not allow discrimination between living and dead biomass, since after a cell dies, amplifiable extracellular DNA can persist in a soil‐like matrix for weeks to years, depending, for instance, on the mineralogy, pH and ionic strength, which can affect the sorption of DNA to the material and prevent its exposure to microbial DNases (Carini *et al*., 2016). Besides, DNA is a molecule that is relatively stable at the temperature required for mushroom cultivation (Bonnet *et al*., 2010). Conversely, PLFAs are structural components of all cells and the phospholipids of dead cells have been shown to be dephosphorylated within minutes in soil (Marschner, 2007) so they can be used as indicators of living biomass, providing a picture of the microbial structure at the time of sampling (García‐Delgado *et al*., 2019).

The content of the V3 and V4 region of the bacterial 16S rRNA gene, quantified by qPCR as a proxy for total bacterial DNA, was scarcely altered during the process of cultivation with similar values registered throughout. However, living bacterial biomass, as measured through PLFA analysis, drastically decreased in phase III and subsequently along the crop cycle after the crop colonization of both substrates, compost and casing. Conversely, the fungal 5.8S rRNA gene content was conditioned by the presence of *A. bisporus* mycelium with low values in non‐colonized compost and high values registered during production. Bacterial alpha‐diversity was reduced after compost colonization (S2 to S3), but increased during cropping (S3 to S4 and S5).Conversely, bacterial alpha‐diversity increased in the colonized casing as previously reported (Carrasco *et al*., 2019). These observations of increasing alpha‐diversity after the application of the casing on top of the compost suggest translation of native bacterial communities from the casing to the compost and from the compost to the casing, configuring a more diverse bacterial microbiome in both substrates.

Beneficial interactions have been described between native bacteria and cultivated mycelium in compost including the hypothetic bacteriolytic enzyme system produced by the champignon that could provide fungi with nutrients from bacteria (Vos *et al*., 2017a; Carrasco and Preston, 2020). This assumption is supported by the microbiome dynamics observed in the compost and casing since the living bacterial biomass assessed by PLFA analysis dropped during the productive stages.

Our results enable us to highlight striking differences between the bacterial communities inhabiting the compost and the casing respectively. The bacterial diversity in compost and casing increased throughout the crop cycle after applying the casing on top of the compost, although the total biomass of bacteria decreased. As reflected by the core microbiome characterization, cased compost (S4 = F1 and S5 = F2) shared high number of OTUs, which indicates microbiota mobility between substrates. *A. bisporus* also possesses its own endophytic microbiota that can contribute to changes observed in the microbiome as it proliferates (Partida‐Martínez, 2017; Zhou *et al*., 2017; Carrasco *et al*., 2019).

The presence of the crop greatly modified the casing microbiome, which is consistent with the enrichment of the surrounding microbiota in substrates colonized by fungal mycelium (Halsey *et al*., 2016; Li *et al*., 2017; Zhou *et al*., 2017). A sharp fall of bacterial biomass, characterized by an increase in alpha‐diversity, was observed, while fungal biomass (as reflected by PLFAs, RT‐qPCR and laccase activity) increased, with a high abundance of living fungal biomass by first flush (F1). Laccase activity in the casing was lower than in compost, which is consistent with the low degradation of lignin and carbohydrate content noticed in the casing.

The techniques employed in this study provide a detailed picture of the changing microbial structure and substrate degradation during the commercial cultivation of champignons. Cultivation of champignons is currently highly dependent on the use of peat for casing material; however, peat reserves are rapidly declining, mainly due to peat mining for agricultural purposes (Peters and von Unger, 2017). The casing material requires specific physicochemical characteristics and a significant microbial load to trigger mushroom fructification (Noble *et al*., 2009; Pardo‐Giménez *et al*., 2017; Zhang *et al*., 2016), but as demonstrated in this study does not contribute substantially to nutrition of the crop. Therefore, understanding of the microbiome composition and dynamics in casing material during commercial cultivation can contribute to efforts to recreate new tailor‐made substrates to provide alternatives to peat. Improvements in the composting process to accelerate compost production and optimize formulation of the mushroom substrate, combined with the design of alternative casing materials, will enhance yields while integrating mushroom cultivation into the circular economy.

## Experimental procedures

### Compost and casing sampling

Samples were destructively taken from the compost at 5 different stages (S1: phase I; S2: phase II; S3: phase III; S4: flush 1; and S5: flush 2) along the composting process and cropping period, and from the peat‐based casing material, Top‐terra^®^ (Legro, Holland), at five successive stages during the cropping cycle (C1: day 1 after the application of the casing; C2: day 6; C3: day 8; C4: flush 1; C5: flush 2). Compost phase I, II and III (spawned with the strain Sylvan A15, Sylvan by the compost yard) was obtained from the same batch from a commercial mushroom compost yard (Germinados de Lodosa, L.S., Lodosa, Spain) and the commercial crop developed at Herchamp S.A.T. (Autol, Spain) (Fig. 1). Approximately 400–500 g of the complete depth of compost or casing was taken randomly from 5 different points of the compost yard and growing room. The beginning and the end of a flush were the first and last day of mushroom picking during the cited flush.

### Metagenomics

Three biological replicates of the compost and casing samples were studied by extracting genomic DNA (*n* = 3 replicates per sample type). Fresh samples were homogenized in a ceramic mortar with liquid nitrogen. DNA was extracted from up to 1 g of compost, or casing respectively with the DNeasy^®^ Power‐Soil^®^ Kit and purified using the DNeasy^®^ PowerClean^®^ Pro Cleanup Kit (Qiagen).

Purified DNA templates were amplified separately for bacteria and fungi by the amplification of the regions of interest through a 2‐step amplification procedure (Rocchi *et al*., 2017; Carrasco *et al*., 2019). The V3 and V4 region of the bacterial 16S rRNA gene was amplified by PCR using paired end universal bacterial primers (Klindworth *et al*., 2013), while the internal transcribed spacer (ITS) was amplified for fungi by employing primers that amplify the ITS2 region, ITS3 and ITS4. Resulting libraries were quantified by Qubit^®^ 3.0 (Thermo Fisher Scientific) and qualified by Fragment Analyzer (Advanced Analytical Technologies) (White *et al*., 1990; Carrasco *et al*., 2019).

PCR amplicons and library fragments were purified, normalized to 4 nM, pooled and denatured with NaOH and diluted with hybridization buffer to 12 pM, using 10% PhiX as a control for low‐diversity libraries (Carrasco *et al*., 2019). Sequencing was performed on an Illumina MiSeq sequencer based in CIBIR (Riojasalud, Gobierno de La Rioja, Spain) using a v3 sequencing kit (2 × 300 cycles) (Illumina, San Diego, CA). The observed cluster density on the flow cell of 690 ± 41 K mm^−2^, with %PF > 88% (number of clusters that passed Illumina’s ‘Chastity filter’) indicated good loading of the library sequenced. An initial average quality control (QC) value of over 30% in > 87% of reads showed good average quality across the whole read length. Data analysis was performed as described by Carrasco *et al*. (2019).

### Phospholipid fatty acids (PLFA) analysis

Microbial population and structure of substrates and casing materials were determined using phospholipid fatty acids (PLFAs) analysis as described in Frostegård *et al*. (1993). Lyophilized samples were extracted with a one‐phase chloroform–methanol–phosphate buffer solvent by sonication. Extracts were purified by SPE, and polar lipids were transesterified with methanol/KOH. Finally, hexane extracts containing the resultant fatty acid methyl esters were analysed by gas chromatography. Quantification was performed using an Agilent 7890 gas chromatograph (Agilent Technologies, Wilmington, DE, USA) equipped with a 25‐mUltra 2 (5% phenyl)‐methylpolysiloxane column (J&W Scientific, Folsom, CA, USA) and a flame ionization detector. PLFAs were identified using bacterial fatty acid standards and software from the microbial Identification System (Microbial ID, Newark, DE, USA). Specific PLFAs (Zelles, 1999) were used as biomarkers to quantify the abundance of Gram‐negative (monounsaturated fatty acids and cyclopropyl 17:0 and 19:0) and Gram‐positive (iso and anteiso saturated branched fatty acids) bacteria, *Actinobacteria* (10‐methyl fatty acids) and fungi (18:2 ω6 cis). Total biomass of bacteria and fungi determined by PLFAs was submitted for the analysis of variance (ANOVA) by previous Levene's variance homogeneity test to determine significant differences between samples. Means were compared by either the Tukey or the Games–Howell post hoc test based on whether or not variance homogeneity was met respectively (*P* < 0.05). ANOVA and post hoc tests were performed using IBM SPSS Statistics v26 software package (IBM SPSS, USA). Principal components analysis (PCA) was performed with PAST v3.15 software (Hammer *et al*., 2001) to compare PLFA composition in substrate and casing samples.

### RT‐qPCR

Extracted genomic DNA (three biological replicates per group) was purified using PowerClean^®^ Pro Cleanup Kit following the manufacturer’s protocol (Qiagen, Hilden, Germany). The DNA was then analysed using an Applied Biosystems StepOnePlusTM Real‐Time PCR system (Thermo Fisher Scientific, Rockford, IL, USA) and the software Applied Biosystems StepOne™ Plus v2.3 software (Thermo Fisher Scientific) for the calculation of Ct values. qPCRBIO SyGreen Mix Hi‐ROX (PCR Biosystems) was used as the intercalating dye for the RT‐qPCR.

Primers designed by Largeteau *et al*. (2007) were employed. The primer pair 5.8S1F (5ʹ‐CAACGGATCTCTTGGCTCT‐3ʹ) and 5.8S2R (5ʹ‐CGCAAGA TGCGTTCAAAGAT‐3ʹ), designed to amplify a 106 bp region of the 5.8S rRNA gene, was used to quantify total fungal DNA. The gene‐specific sequences used for bacterial DNA quantification, S‐D‐Bact‐0341‐b‐S‐17, 5′‐CCTACGGGNGGCWGCAG‐3′ and S‐D‐Bact‐0785‐a‐A‐21, 5′‐GACTACHVGGGTATCTAATCC‐3, target the 16S rRNA V3‐V4 region (Herlemann *et al*., 2011). A two‐step RT‐qPCR was performed with cycling conditions of holding stage 95°C for 10 min, followed by cycling stage of 40 cycles at 95°C for 15 s and 60°C for 1 min. Melt curve analysis was performed at the end of each PCR run to test for the presence of a unique PCR product. A melt curve profile was obtained by heating the mixture to 95°C (15 s, step 1), cooling to 60°C (1 min, step 2) and slowly heating to 95°C (15 s) at 0.3°C s^–1^ (step 3) with continuous measurement of fluorescence at 520 nm. A derivative report from the melt curve was implemented using Applied Biosystems StepOne™ Plus v2.3 software (Thermo Fisher Scientific) to check the robustness of the analysis (Fig. S5).

### Laccase activity measurement and calculation

The extraction of laccase followed the method described by Vos *et al*. (2017a). Each sample was prepared for laccase extraction by homogenization after flash freezing in a −80°C freezer followed by lyophilization and milling. Laccase activity was determined using the rate of oxidation of 2,2ʹ‐azino‐bis‐ethylbenzothiazoline‐6‐sulfonic acid (ABTS) measured by spectrophotometry at *λ* = 420 nm. Commercial laccase from *Trametes versicolor* (Merck, Germany) was used to generate the calibration curve. ABTS solution was prepared by diluting ABTS powder in citric phosphate buffer (pH = 4) to a final concentration of 1 mM was used to blank the spectrophotometer. Incubation of samples for laccase extraction was performed by mixing 50 mg sample with 1 ml of sterile Milli‐Q water in a 2 ml Eppendorf tube by incubating with continuous rotation for 1 h at 25°C. Insoluble material was removed by centrifugation at 4°C and 15 000 *g* for 15 min. Twenty microlitres of the supernatant from each sample was added to 980 μl of 1 mM ABTS. ABTS oxidation rate was monitored by determining absorbance variability at *λ* = 420 nm for 30 s.

The activity and therefore the rate of ABTS oxidation due to the extracellular laccase in each sample was calculated using the Beer–Lambert law with an extinction factor of 36 000 M^−1^cm^−1^. Laccase activity was expressed in units defined as 1 U = 1 µmol of ABTS oxidized per min. Final data were modified to show enzyme activity per gram of sample (U g^−1^).

### Quantification of lignin and complex carbohydrates

The procedure for determination of neutral detergent fibre (NDF), acid detergent fibre (ADF) and lignin from dry compost and casing used was adapted from the methodology described by Van Soest *et al*. (1991). Five analytical replicates per sample were evaluated. For the quantification of the lignin and carbohydrate content, a Fibertec™ 8000 (Foss) was employed. The amount of cellulose, hemicellulose, lignin and other macromolecules (such as cutin) was initially calculated from 0.5–1 g of dry sample (W_1_; dm = dry matter) prepared in a crucible. To estimate the amount of NDF, samples were dissolved in 100 ml of neutral detergent solution and 4–5 drops of antifoam agent (1‐octanol) (Van Soest *et al*., 1991). Samples were subsequently subjected to vacuum filtration, washed 3 times with distilled water and finally with acetone. Dry samples were weighed (W_2_) and calcinated in a muffle furnace at 520°C (W_3_). The proportion of the original sample corresponding to NDF was calculated using the following equation:

$$\%\;\text{NDF}\;\text{dm}=[\left(W_{2}‐W_{3}\right)/W_{1}]/f*100$$

f=(1‐H/100);H:%of moisture in the sample

ADF was calculated from 0.5–1 g of dry sample (W_4_) dissolved in 100 ml of acid detergent solution and 4–5 drops of antifoam agent (1‐octanol) (Van Soest *et al*., 1991) as described above. The dry sample (W_5_) was subsequently calcinated (W_6_). This process dissolves hemicellulose and allows cellulose, lignin and cutin content to be estimated using the following equation:

$$\%\;\text{NDF}\;\text{dm}=[\left(W_{5}‐W_{6}\right)/W_{4}]/f*100$$

Finally, the ADF residue was digested with concentrated sulfuric acid (72%) to degrade cellulose and cutin (W_7_). The organic fraction remaining corresponding to the lignin content was calculated following calcination of this sample (W_8_).

$$\%\;\text{NDF}\;\text{dm}=[\left(W_{7}‐W_{8}\right)/W_{1}]/f*100$$

### Statistical analysis of RT‐qPCR, laccase analysis and structural composition

Statistical analysis was performed using Statgraphics Centurion XVII software. Different outputs were compared by analysis of variance (ANOVA) followed by, depending on whether the assumption of normality and homogeneity of variance were met, post hoc Tukey's test (at 5% probability) or non‐parametric tests, including the Kruskal–Wallis test and the Mann–Whitney (Wilcoxon) W test to compare medians at the 95% level for non‐normal distributions.

## Conflict of interest

There are no conflicts of interests.

## Supporting information


**Table S1.** Library size after cleaning sequences. *Groups corresponding to: Compost (S1: phase I, S2: phase II and S3: phase III, S4: flush 1 and S5: flush 2) and casing material (C1: day 1, C2: day 6, C3: day 8, C4: flush 1 and C5: flush 2).
**Fig. S1.** Heatmap summarizing the hierarchy of microbiome components: (a) based on the bacteria phyla and (b) based on fungi phyla. The *x*‐axis corresponds to the clustering of the different samples (groups and single samples), while the y‐axis corresponds to the clustering of the most abundant OTUs (97% similarity) among reads. The distance between data in the clustering input (Ward algorithm for analysis of variance implemented) has been calculated with Euclidean parameters. Compost (S1: phase I, S2: phase II and S3: phase III, S4: flush 1 and S5: flush 2) and casing material (C1: day 1, C2: day 6, C3: day 8, C4: flush 1 and C5: flush 2).
**Fig. S2.** The bacterial microbiome of basidiomes is similar in composition to the casing microbiome. (a) Comparison of the identified OTU components of the bacterial microbiome along the crop cycle in the compost. (b) Comparison of the identified OTU components of the fungal microbiome along the crop cycle in the compost. (c) Comparison of the identified OTU components of the bacterial microbiome along the crop cycle in the casing. (d) Comparison of the identified OTU components of the fungal microbiome along the crop cycle in the casing. Compost (S1: phase I, S2: phase II and S3: phase III, S4: flush 1 and S5: flush 2) and casing materials (C1: day 1, C2: day 6, C3: day 8, C4: flush 1 and C5: flush 2). Overlapping areas in Venn diagrams represent shared elements. The open‐source Metagenomics Core Microbiome Exploration Tool (MetaCoMET) (USDA, USA) was used to compare the core microbiome (Wang *et al*., 2016).
**Fig. S3.** Network analysis revealing co‐occurrence patterns among sample groups of compost (a, b) and casing (c, d): (a) Bacterial phyla identified in compost samples; (b) Fungal genera identified in compost samples; (c) Bacterial phyla identified in casing samples; (d) Fungal genera identified in casing samples. The nodes were coloured according to modularity class. A connection in red represents a positive strong (Pearson correlation coefficient *ρ* > 0.8) and significant (*P*‐value < 0.01) correlation while a connection in blue represents a negative correlation. The size of each node is proportional to the number of connections. Compost (S1: phase I, S2: phase II and S3: phase III, S4: flush 1 and S5: flush 2) and casing material (C1: day 1, C2: day 6, C3: day 8, C4: flush 1 and C5: flush 2).
**Fig. S4.** Biomass of (a) Gram‐, (b) Gram+ and (c) *Actinobacteria*, quantified by PLFA analysis. Bars indicate standard deviation (*n* = 4). Different letters indicate significant differences between samples (*P* < 0.05). Compost (S1: phase I, S2: phase II and S3: phase III, S4: flush 1 and S5: flush 2) and casing material (C1: day 1, C2: day 6, C3: day 8, C4: flush 1 and C5: flush 2).
**Fig. S5.** Melt curve analysis was performed at the end of each PCR run to test for the presence of a unique PCR reaction product. (a) V3‐V4 16S rRNA (bacteria); (b) 5.8S rRNA (fungi). Applied Biosystems StepOnePlus™ Instrument.

**Data S1.** Data set generated for the PLFAs analysis described in this project: normalized data corresponding to the different replicates per group of samples and the values of each variable (organism) used to create the PCA (Figure 5).

## Data Availability

Data associated with the metataxonomic analysis (16S rRNA for bacteria and ITS2 region between 5.8S and 28S rRNA for fungi; including the biom file and metadata table for each data set) described in this project have been uploaded in Oxford Research Archives (ORA) and can be downloaded from this link: https://ora.ox.ac.uk/objects/uuid:800d1b34‐6c76‐4099‐b6f9‐8a699fb53e5e.
