# Supplementary material for: Holistic assessment of the microbiome dynamics in the substrates used for commercial champignon (Agaricus bisporus) cultivation
Source: Microb Biotechnol. 2020 Jul 27;13(6):1933–47. doi: 10.1111/1751-7915.13639 (PMC7533343; doi:10.1111/1751-7915.13639)
Supplement: Supplementary file 1 — Table S1. Library size after cleaning sequences. *Groups corresponding to: Compost (S1: phase I, S2: phase II and S3: phase III, S4: flush 1 and S5: flush 2) and casing material (C1: day 1, C2: day 6, C3: day 8, C4: flush 1 and C5: flush 2). Fig. S1. Heatmap summarizing the hierarchy of microbiome components: (a) based on the bacteria phyla and (b) based on fungi phyla. The x‐axis corresponds to the clustering of the different samples (groups and single samples), while the y‐axis corresponds to the clustering of the most abundant OTUs (97% similarity) among reads. The distance between data in the clustering input (Ward algorithm for analysis of variance implemented) has been calculated with Euclidean parameters. Compost (S1: phase I, S2: phase II and S3: phase III, S4: flush 1 and S5: flush 2) and casing material (C1: day 1, C2: day 6, C3: day 8, C4: flush 1 and C5: flush 2). Fig. S2. The bacterial microbiome of basidiomes is similar in composition to the casing microbiome. (a) Comparison of the identified OTU components of the bacterial microbiome along the crop cycle in the compost. (b) Comparison of the identified OTU components of the fungal microbiome along the crop cycle in the compost. (c) Comparison of the identified OTU components of the bacterial microbiome along the crop cycle in the casing. (d) Comparison of the identified OTU components of the fungal microbiome along the crop cycle in the casing. Compost (S1: phase I, S2: phase II and S3: phase III, S4: flush 1 and S5: flush 2) and casing materials (C1: day 1, C2: day 6, C3: day 8, C4: flush 1 and C5: flush 2). Overlapping areas in Venn diagrams represent shared elements. The open‐source Metagenomics Core Microbiome Exploration Tool (MetaCoMET) (USDA, USA) was used to compare the core microbiome (Wang et al., 2016). Fig. S3. Network analysis revealing co‐occurrence patterns among sample groups of compost (a, b) and casing (c, d): (a) Bacterial phyla identified in compost samples; (b) Fungal genera ident [file MBT2-13-1933-s001.docx]

**[Holistic assessment of the microbiome dynamics](https://msystems.asm.org/content/3/4/e00038-18.short) in the substrates used for commercial champignon (*Agaricus bisporus*) cultivation**

**CARRASCO Jaime^1,2*^, GARCÍA-DELGADO Carlos^3,4^, LAVEGA Rebeca^2^, TELLO María L.^2^, DE TORO María^5^, BARBA-VICENTE Víctor^4^, RODRÍGUEZ-CRUZ María S.^4^, SÁNCHEZ-MARTÍN María J.^4^, PÉREZ Margarita^2^, PRESTON Gail M.^1^**

^1^Department of Plant Sciences, University of Oxford, S Parks Rd, Oxford OX1 3RB, UK; ^2^Centro Tecnológico de Investigación del Champiñón de La Rioja (CTICH), Autol, Spain; ^3^Departamento de Geología y Geoquímica, Universidad Autónoma de Madrid, Spain; ^4^Institute of Natural Resources and Agrobiology of Salamanca (IRNASA-CSIC), Salamanca, Spain; ^5^Plataforma de Genómica y Bioinformática, Centro de Investigación Biomédica de La Rioja (CIBIR), Logroño, Spain.

^*^Corresponding author: carraco.jaime@gmail.com; jaime.carrasco@plants.ox.ac.uk.

**SUPPORTING INFORMATION**

**Table S1.** Library size after cleaning sequences.

*Groups corresponding to: Compost (S1: phase I, S2: phase II and S3: phase III, S4: flush 1 and S5: flush 2) and casing material (C1: day 1, C2: day 6, C3: day 8, C4: flush 1 and C5: flush 2).


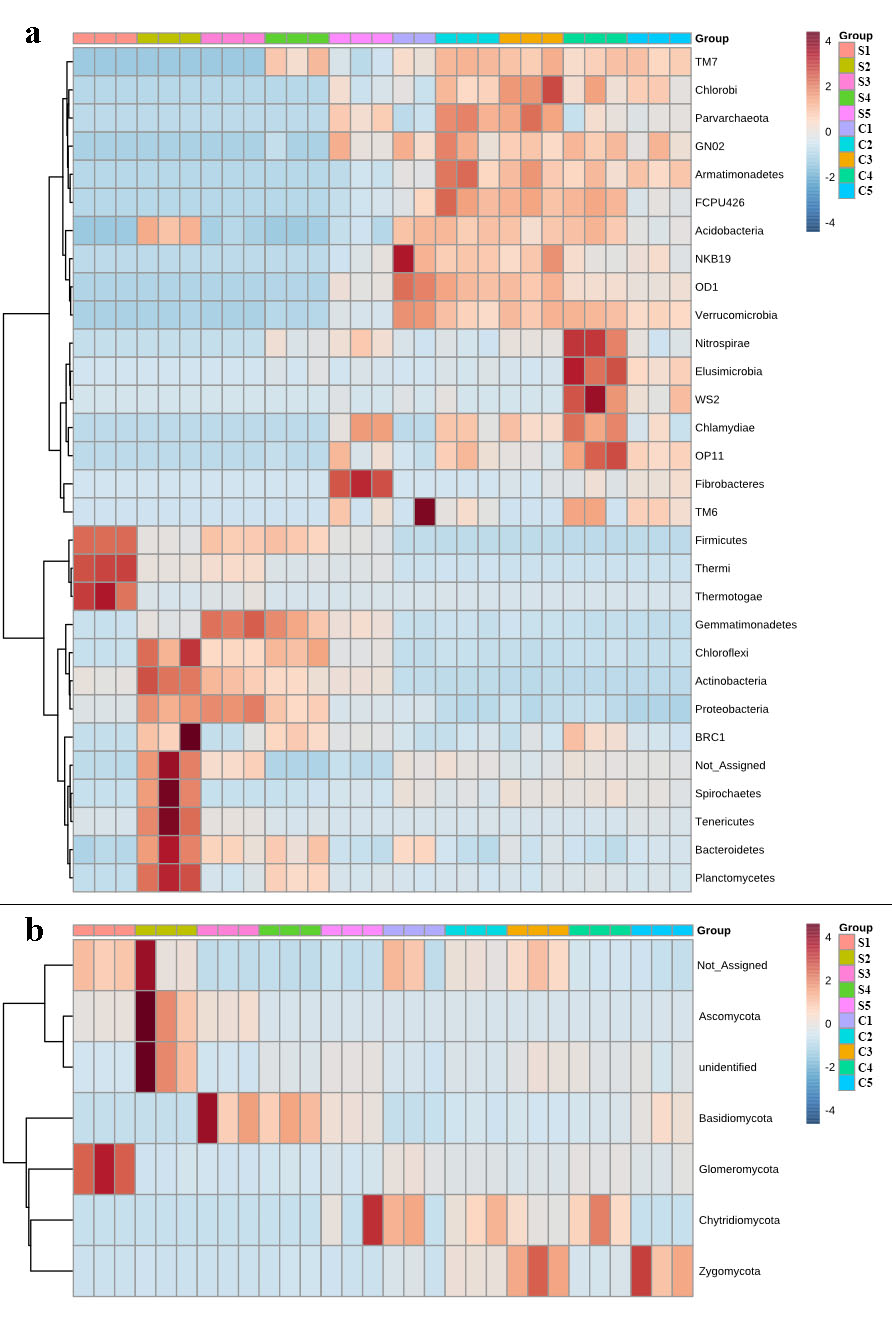


**Fig. S1.** Heatmap summarising the hierarchy of microbiome components: (a) based on the bacteria phyla and (b) based on fungi phyla. The x-axis corresponds to the clustering of the different samples (groups and single samples), while the y-axis corresponds to the clustering of the most abundant OTUs (97 % similarity) among reads. The distance between data in the clustering input (Ward algorithm for analysis of variance implemented) has been calculated with Euclidean parameters. Compost (S1: phase I, S2: phase II and S3: phase III, S4: flush 1 and S5: flush 2) and casing material (C1: day 1, C2: day 6, C3: day 8, C4: flush 1 and C5: flush 2).


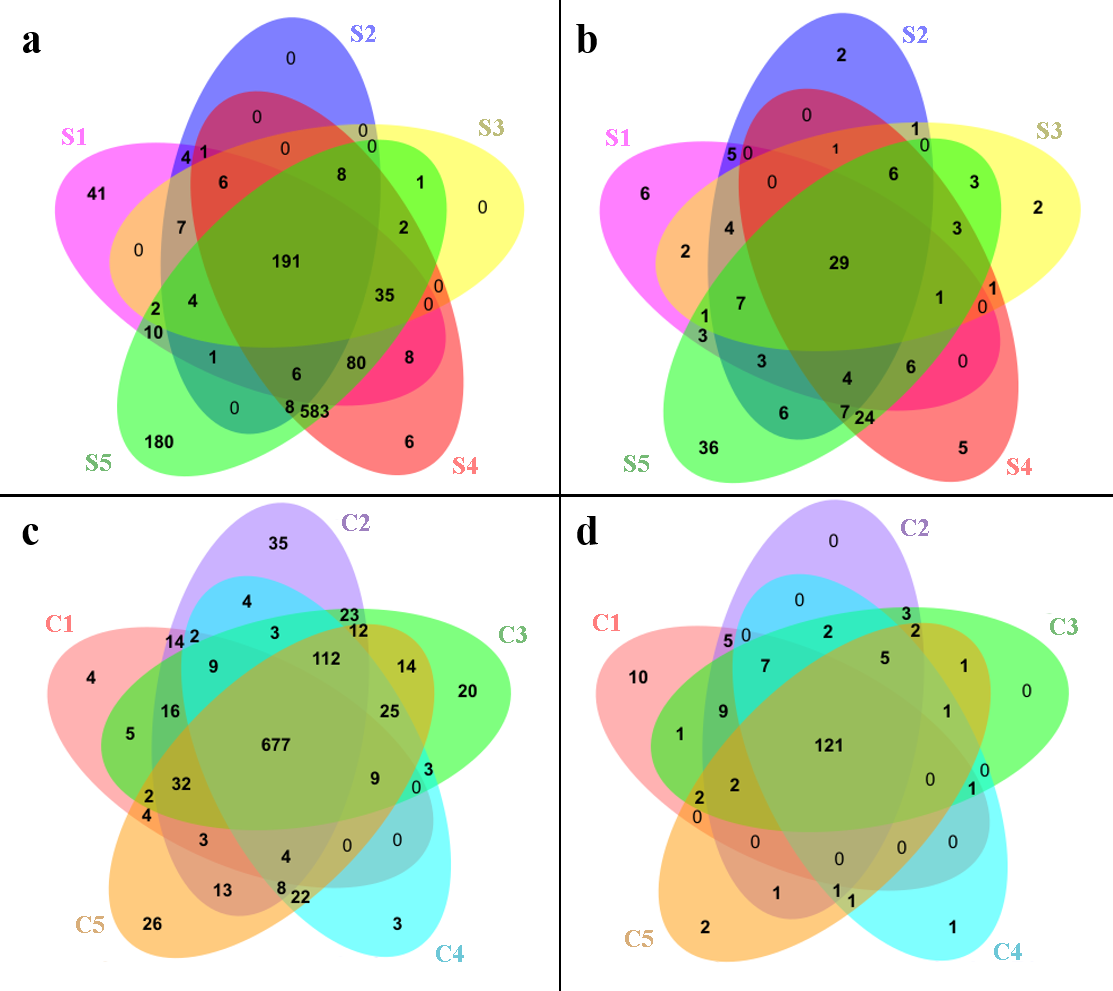


**Fig. S2.** The bacterial microbiome of basidiomes is similar in composition to the casing microbiome. (a) Comparison of the identified OTU components of the bacterial microbiome along the crop cycle in the compost. (b) Comparison of the identified OTU components of the fungal microbiome along the crop cycle in the compost. (c) Comparison of the identified OTU components of the bacterial microbiome along the crop cycle in the casing. (d) Comparison of the identified OTU components of the fungal microbiome along the crop cycle in the casing. Compost (S1: phase I, S2: phase II and S3: phase III, S4: flush 1 and S5: flush 2) and casing materials (C1: day 1, C2: day 6, C3: day 8, C4: flush 1 and C5: flush 2). Overlapping areas in Venn diagrams represent shared elements. The open-source Metagenomics Core Microbiome Exploration Tool (MetaCoMET) (USDA, USA) was used to compare the core microbiome (Wang *et al.*, 2016).

**Fig. S3.** Network analysis revealing co-occurrence patterns among sample groups of compost (a, b) and casing c, d): a) Bacterial phyla identified in compost samples; b) Fungal genera identified in compost samples; c) Bacterial phyla identified in casing samples; d) Fungal genera identified in casing samples. The nodes were coloured according to modularity class. A connection in red represents a positive strong (Pearson correlation coefficient ρ>0.8) and significant (P-value <0.01) correlation while a connection in blue represents a negative correlation. The size of each node is proportional to the number of connections. Compost (S1: phase I, S2: phase II and S3: phase III, S4: flush 1 and S5: flush 2) and casing material (C1: day 1, C2: day 6, C3: day 8, C4: flush 1 and C5: flush 2).

**c**

d

d

d

d

b

cd

cd

b

a

c

e

e

e

e

c

e

e

d

a

b

**b**

**a**

e

e

e

e

e

e

b

c

a

d


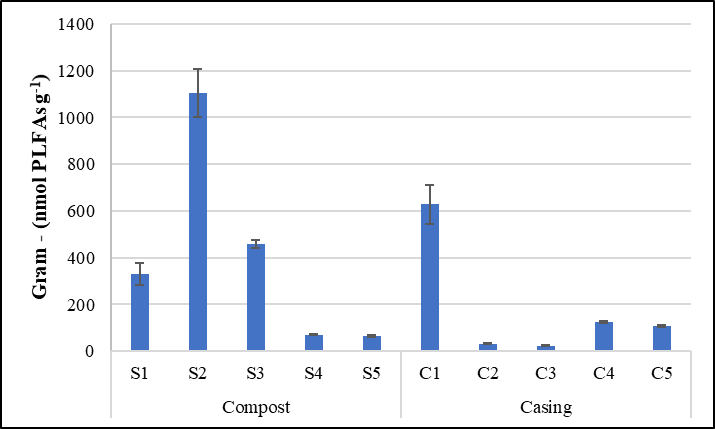

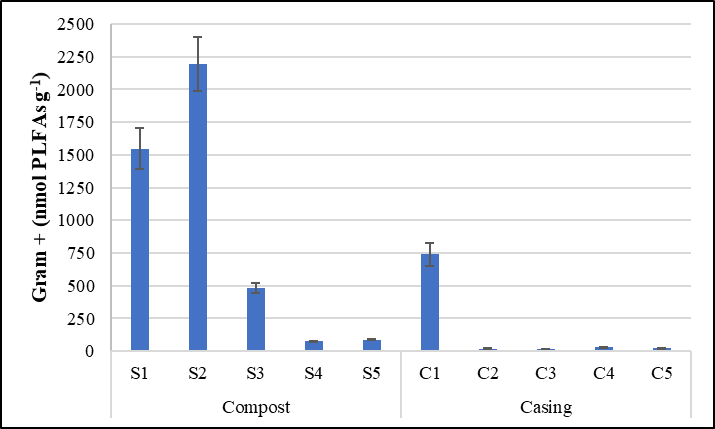

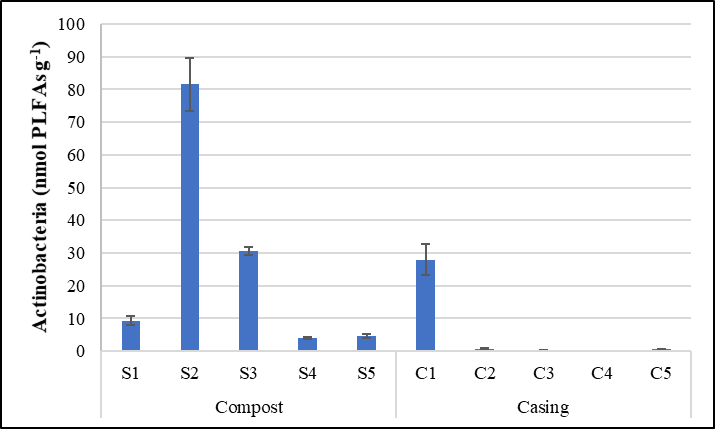


**Fig. S4.** Biomass of a) Gram-, b) Gram+ and c) *Actinobacteria*, quantified by PLFA analysis. Bars indicate standard deviation (n = 4). Different letters indicate significant differences between samples (*p* < 0.05). Compost (S1: phase I, S2: phase II and S3: phase III, S4: flush 1 and S5: flush 2) and casing material (C1: day 1, C2: day 6, C3: day 8, C4: flush 1 and C5: flush 2).

**Fig. S5.** Melt curve analysis was performed at the end of each PCR run to test for the presence of a unique PCR reaction product. a) V3-V4 16S rRNA (bacteria); b) 5.8S rRNA (fungi). Applied Biosystems StepOnePlus™ Instrument.
